# Supplementary figures and images for: Adaptation and potential culture of wild Amphipods and Mysids as potential live feed in aquaculture: a review
Source: PeerJ. 2024 Mar 29;12:e17092. doi: 10.7717/peerj.17092 (PMC10984187; doi:10.7717/peerj.17092)

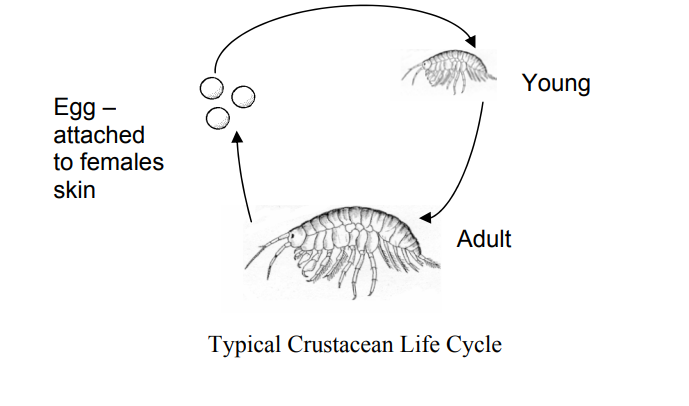

Supplement: Supplemental Information 1 [file peerj-12-17092-s001.png]

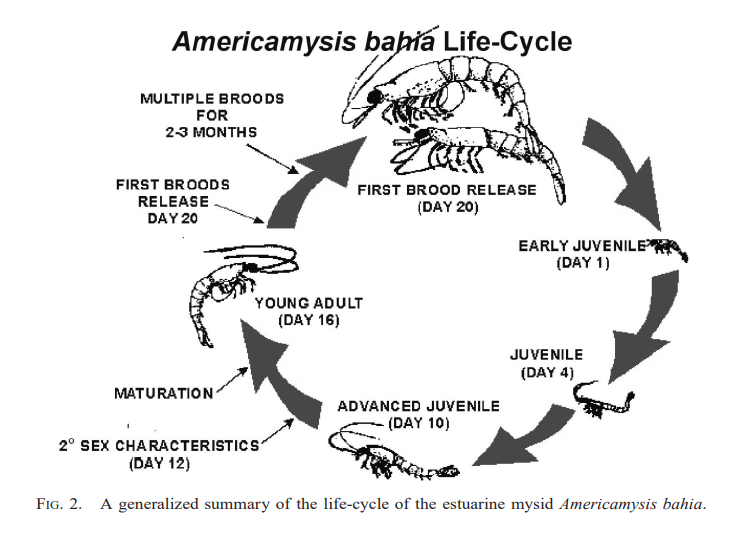

Supplement: Supplemental Information 2 [file peerj-12-17092-s002.png]
